# The Menstrual Distress Questionnaire (MEDI-Q): reliability and validity of the German version

**DOI:** 10.1186/s12905-026-04608-7

**Published:** 2026-07-14

**Authors:** Yvonne Görlich, Elena Richter

**Affiliations:** Department of Psychology, PFH Private Hochschule Göttingen, PFH Private University of Applied Sciences, Weender Landstraße 3-7, Göttingen, 37073 Germany

**Keywords:** Menstrual distress, Dysmenorrhea, Premenstrual symptoms, MEDI-Q, Psychometrics, Confirmatory factor analysis, Cross-cultural validation

## Abstract

**Background:**

Menstrual distress affects a substantial proportion of individuals of reproductive age and is associated with impaired quality of life and psychological comorbidities, yet validated German-language instruments for its assessment are lacking. The present study examined the reliability and validity of the German version of the Menstrual Distress Questionnaire (MEDI-Q), a 25-item self-report instrument previously validated in Italian, English, and Indonesian.

**Methods:**

A community sample of *N* = 1,035 German-speaking women completed the MEDI-Q alongside the DASS-21, the PAF10, and a global rating of menstrual symptom severity. Internal consistency, test-retest reliability, convergent validity, and structural validity via confirmatory factor analysis (CFA) were examined.

**Results:**

Internal consistency was high (α = 0.839, ω = 0.844) and test-retest reliability acceptable over a naturalistic long-term interval (ICC = 0.823). MEDI-Q Total Score, MS, and MSD correlated negatively with age and positively with psychological distress, premenstrual symptoms, and global symptom severity, while the Menstrual Specificity Index (MESI) showed a significantly weaker and statistically distinct association with the global rating. Among three competing factor models, the theoretically proposed 5-factor structure showed the best relative fit, though none of the models reached conventional fit thresholds (CFI/TLI ≥ 0.90), and structural validity warrants further investigation. Applying the proposed cut-off of ≥ 20, 36.4% of the community sample exceeded this threshold.

**Conclusions:**

The German MEDI-Q represents a psychometrically adequate instrument for standardised assessment of menstrual distress in German-speaking contexts, facilitating cross-cultural comparisons across four language versions, while structural validity remains unestablished and the instrument should be interpreted as a total score measure pending further investigation.

**Supplementary Information:**

The online version contains supplementary material available at 10.1186/s12905-026-04608-7.

## Introduction

Menstruation is a universal biological experience across the reproductive lifespan, involving cyclical hormonal fluctuations that exert broad effects on emotional processing, cognitive functioning, mood, sleep, appetite, and pain sensitivity [1–3]. For a substantial proportion of women, these cyclical changes manifest as clinically significant menstrual complaints that impair daily functioning and reduce quality of life.

Epidemiological data underscore the considerable burden of menstrual complaints. A large-scale Dutch survey (*N* > 42,000) reported that 85% of women experienced lower abdominal pain during menstruation, with 38% unable to fulfil usual daily obligations [4]. German population data indicate that approximately 23.7% of women aged 14–55 reported impairing menstrual complaints [5]. A recent meta-analysis of 336 studies estimated the pooled worldwide prevalence of dysmenorrhoea at 71.3% [6], with severe cases significantly impacting school and work attendance [7, 8].

Menstrual distress refers to the negative, subjectively experienced burden resulting from menstruation-related stressors. Elevated menstrual distress has been associated with reduced quality of life [9], comorbid gynaecological conditions including endometriosis and heavy menstrual bleeding [10, 11], and a well-established bidirectional relationship with psychological distress: stress and negative affect both exacerbate and result from menstrual complaints [12–15].

### Existing measurement instruments and their limitations

Despite the clinical and public health relevance of menstrual distress, validated psychometric instruments specifically targeting the menstrual phase remain limited. The Menstrual Distress Questionnaire (MDQ [16]) was among the earliest standardised tools, but subsequent evaluations identified limitations in internal consistency and factorial validity [17]. The Menstrual Bleeding Questionnaire [18] narrows its scope to blood loss. The Daily Symptom Rating Scale [19] requires prospective daily diary completion across one cycle, raising feasibility concerns, and has demonstrated uncertain factor structure [17]. More broadly, existing instruments tend to focus predominantly on the premenstrual phase, with comparatively little attention to the intramenstrual period. Premenstrual Syndrome (PMS) and its severe form, Premenstrual Dysphoric Disorder (PMDD), have received considerable research attention and are assessed by instruments including the Daily Record of Severity of Problems [20]. However, these instruments specifically target the luteal phase and are not designed to capture distress experienced during menstruation proper. The MEDI-Q fills this conceptual gap by focusing explicitly on the menstrual phase, providing a complementary assessment perspective. This phase-specific focus is clinically meaningful, as menstrual and premenstrual complaints are conceptually and empirically distinct, and individuals may present with primarily intramenstrual complaints without meeting criteria for PMS or PMDD.

### The Menstrual Distress Questionnaire (MEDI-Q)

The MEDI-Q was originally developed and validated in Italian [21], and is designed to comprehensively assess menstrual distress during the menstrual days proper. The instrument covers 25 symptoms across five domains: pain, gastrointestinal symptoms, discomfort, psychological and cognitive changes, and other physiological changes.

For each symptom, respondents rate (A) frequency over the past 12 menstrual cycles and, if present, the degree of impairment to quality of life and daily activities (B) during menstruation, (C) during the premenstrual phase, and (D) during the intermenstrual phase. The instrument yields four composite indices: the MEDI-Q Total Score (range 0–125), Menstrual Symptoms (MS; range 0–25), Menstrual Symptom Distress (MSD; range 0–5), and the Menstrual Specificity Index (MESI; range 0–1).

The Italian original demonstrated excellent psychometric properties (α = 0.85, ICC = 0.95 [21]). An English-language validation replicated these findings (α = 0.84, ICC = 0.95 [22]), and an Indonesian validation further corroborated the instrument’s cross-cultural robustness (α = 0.972, ICC = 0.892 [23]). The present study constitutes the fourth language validation of the MEDI-Q.

### The need for a German validation

German-speaking countries (Germany, Austria, Switzerland) constitute a population exceeding 100 million individuals with a well-developed healthcare research infrastructure. The absence of a validated German-language menstrual distress instrument represents a meaningful gap, as existing German-language tools focus predominantly on premenstrual symptoms (e.g., PAF10 [24]) or general symptom checklists not specific to the menstrual phase. Cultural and linguistic adaptation requires rigorous validation to ensure construct equivalence, as failure to establish equivalence risks systematic over- or underestimation of construct severity due to cultural differences in symptom labelling or response tendencies [25–27].

Moreover, menstruation remains a culturally sensitive topic associated with stigma and shame across Western societies, reinforcing communication taboos and contributing to delayed help-seeking [28–30]. This pattern extends to German-speaking contexts, where menstrual stigma and concealment norms have been documented in both public discourse and clinical settings [4, 31].

### Hypotheses

The present study examined the reliability and validity of the German version of the MEDI-Q. Both prior Western validations demonstrated high internal consistency (α = 0.85 and α = 0.84, respectively) and excellent test-retest reliability (ICC = 0.95 in both studies [21, 22]).

- *H1a: The German MEDI-Q demonstrates high internal consistency (Cronbach’s α ≥ 0.80 and McDonald’s ω ≥ 0.80).*
- *H1b: The German MEDI-Q demonstrates acceptable test-retest reliability (ICC ≥ 0.70) for the MEDI-Q Total Score.*

Several studies have reported that menstrual symptom severity declines with advancing reproductive age [21, 32, 33].

- *H2: MEDI-Q subscale scores (Total Score*,* MS*,* MSD*,* MESI) correlate negatively with age.*

A robust association between menstrual symptom burden and general psychological distress has been documented across multiple study designs [12–14, 34], and was replicated in both prior MEDI-Q validations [21, 22].

- *H3: MEDI-Q Total Score*,* MS*,* and MSD correlate positively with DASS-21 Depression*,* Anxiety*,* and Stress subscale scores.*

Premenstrual and menstrual symptoms frequently co-occur; as early as 1953, Greene and Dalton observed that similar symptoms occasionally extend into the early days of menstruation [35]. This phase overlap is reflected in the multi-phase structure of the MEDI-Q [21, 22].

- *H4: MEDI-Q Total Score*,* MS*,* and MSD correlate positively with PAF10 total scores.*

The MESI captures symptoms uniquely aggravated during menstruation relative to both the premenstrual and intermenstrual phases. Individuals with high premenstrual symptom burden (high PAF10) would be expected to show lower MESI scores, as their symptoms are not restricted to the menstrual phase but extend into the premenstrual phase, thereby increasing the denominator of the MESI ratio and reducing the index of phase-specificity – a pattern confirmed in the Italian validation [21].

- *H5: MEDI-Q MESI correlates negatively with PAF10 total scores.*

Global single-item ratings show adequate convergent validity with multi-item scales [36, 37]. Total Score, MS, and MSD are expected to correlate positively with a global menstrual symptom rating. The MESI, reflecting phase-specificity rather than overall severity, is expected to show a significantly weaker association.

- *H6a: MEDI-Q Total Score is positively associated with a single-item global rating of menstrual symptom severity.*
- *H6b: MEDI-Q MS is positively associated with a single-item global rating of menstrual symptom severity.*
- *H6c: MEDI-Q MSD is positively associated with a single-item global rating of menstrual symptom severity.*
- *H6d: The association between MEDI-Q MESI and a single-item global rating of menstrual symptom severity is significantly weaker (small effect r < .30) compared to the associations observed for MEDI-Q Total Score, MS, and MSD (medium to large effects,r ≥ .30).*

In addition to these convergent validity hypotheses, the present study provides the first examination of the latent factor structure of the MEDI-Q using confirmatory factor analysis, comparing three theoretically motivated competing models. As no prior validation has included factor-analytic examination of the instrument, no directional hypothesis regarding model fit is specified; the analyses are intended to evaluate the plausibility of the theoretically proposed domain structure and to inform future structural refinement of the MEDI-Q.

## Method

### Translation and cultural adaptation

Translation followed a rigorous multi-stage forward-backward procedure in accordance with ITC Guidelines [38] and established recommendations [26, 39]. Written permission was obtained from the lead author of the Italian original. The English-language version [22] served as the primary source text; the Italian original [21] was consulted throughout to ensure fidelity to the source instrument. Two independent German native speakers produced separate forward translations (Stage 1), which were subsequently reconciled item by item until consensus was reached (Stage 2). The reconciled version was back-translated into Italian and English by two further independent translators; any discrepancies were resolved by the original translators in reference to the Italian source, which was considered the more critical verification (Stage 3). A German native-speaking woman from the target population confirmed comprehensibility in a final face validity check (Stage 4); no amendments were required. The German version of the MEDI-Q is provided in Supplementary Material 1, Appendix A.

### Sample

Eligibility criteria required participants to be female, aged 18–50 years, currently menstruating, and to have experienced at least three menstrual cycles in the preceding 12 months. Women with diagnosed psychiatric disorders or uterine pathology (e.g., fibroids, endometriosis) were excluded. These criteria align with those employed in the Italian original study [21].

The final sample comprised *N* = 1,035 women. Participants ranged in age from 18 to 50 years (*M* = 31.63, *SD* = 7.07), with a mean age at menarche of 13.08 years (*SD* = 1.44, range 9–19). Mean menstrual cycle length was 28.64 days (*SD* = 3.51, range 17–40), and mean menstruation duration was 4.93 days (*SD* = 1.25, range 1–10). The majority of participants resided in Germany (*n* = 888, 85.8%), followed by Switzerland (*n* = 56, 5.4%), Austria (*n* = 55, 5.3%), and other countries (*n* = 36, 3.5%). Hormonal contraceptive use was reported by 126 participants (12.2%). Hormonal contraceptive users were not excluded, as the MEDI-Q is designed to assess subjective menstrual distress regardless of hormonal context; their inclusion reflects the instrument’s intended use in population-level research and assessment.

### Measures

#### Menstrual distress

The 25-item MEDI-Q assesses menstrual symptom frequency (scored 0–2) and distress during menstrual, premenstrual, and intermenstrual phases (scored 0–3 per phase). Composite indices (Total Score, MS, MSD, MESI) were computed according to the published scoring algorithm [21, 22]. A Total Score ≥ 20 has been proposed as a cut-off for clinically relevant menstrual distress.

#### Depression, anxiety, and stress

The Depression Anxiety Stress Scales–21 (DASS-21 [40, 41]) comprises three seven-item subscales rated on a four-point scale. Internal consistency in German samples was α ≥ 0.91 (Depression), α = 0.78–0.82 (Anxiety), and α = 0.81–0.89 (Stress) [41].

### Premenstrual symptoms

The Premenstrual Assessment Form–10 (PAF10 [24, 42]) is a 10-item retrospective measure of premenstrual psychological and physiological symptoms (total score 0–50; ≥ 23 indicates clinical relevance; α = 0.88). The PAF10 was also used in the Italian and English MEDI-Q validations [21, 22], enabling direct cross-study comparison.

### Global rating of menstrual symptom severity

Participants rated average menstrual symptom intensity on a single-item numerical rating scale (1 = no symptoms to 10 = extremely severe). Single-item global ratings correlate adequately with multi-item scales and represent a valid, efficient measure of subjective symptom burden [36, 37].

### Procedure

The study was conducted as an anonymous online survey using LimeSurvey. Participants were recruited through online channels using convenience and snowball sampling. After providing informed consent, participants completed the questionnaires in fixed order: MEDI-Q, DASS-21, PAF10, and global symptom rating. Demographic and menstrual history items were collected at the beginning of the survey. The study was approved by the Ethics Committee of PFH Private University of Applied Science and conducted in accordance with the Declaration of Helsinki.

A subset of participants (*n* = 20) voluntarily completed the MEDI-Q a second time to allow for an exploratory assessment of test-retest reliability. The interval between the two assessments ranged from 144 to 280 days (*M* = 180.50, *SD* = 36.87).

### Data analysis

All statistical analyses were conducted in IBM SPSS Statistics (Version 29); confirmatory factor analyses were performed in IBM AMOS (Version 29). Differences in sample size across analyses reflect missing data on individual measures; all available data were used for each analysis.

Internal consistency was evaluated using Cronbach’s α and McDonald’s ω [43], accompanied by corrected item-total correlations as indicators of item discrimination. Although α remains widely used, it assumes tau-equivalence across items, that is, equal factor loadings, an assumption that is unlikely to hold for an instrument covering a heterogeneous symptom spectrum. McDonald’s ω relaxes this assumption and provides a more appropriate model-based reliability estimate when item loadings vary [44].

Test-retest reliability was assessed using the intraclass correlation coefficient (ICC; two-way mixed-effects model, absolute agreement), consistent with prior MEDI-Q validations [21, 22], with ICC ≥ 0.70 defined as acceptable. Given the small subsample size (*n* = 20) and naturalistic retest interval, ICC results are considered exploratory.

Although no prior validation of the MEDI-Q has included factor-analytic examination, we proceeded directly to CFA rather than exploratory factor analysis (EFA). This decision was motivated by the existence of a theoretically specified five-domain structure proposed by the instrument’s developers [21], which provided sufficient a priori justification for confirmatory testing. The instrument’s heterogeneous symptom content would be expected to produce ambiguous EFA solutions without theoretical guidance. Maximum likelihood estimation was employed consistent with the interval-level scaling assumptions underlying the MEDI-Q scoring algorithm. Three competing models were specified: a single-factor model, a two-factor model distinguishing psychological from physiological symptoms, and a five-factor model based on the theoretical domain structure proposed by Vannuccini et al. [21]. Model fit was evaluated using CFI, TLI, and RMSEA (with 90% CI), with acceptable fit defined as CFI and TLI ≥ 0.90, and RMSEA ≤ 0.08 [45]. Model comparison used the chi-square difference test and AIC.

Convergent validity was assessed using Pearson correlations (two-tailed) between all four MEDI-Q indices and age, DASS-21 subscales, PAF10 scores, and a global symptom severity rating. Effect sizes were interpreted using Cohen’s benchmarks [46]. Differences between dependent correlation coefficients (H6d) were tested using Steiger’s Z-test [47]. A priori power analysis (G*Power 3.1) indicated *N* = 134 as the minimum to detect *r* = .30 with 95% power (α = 0.05); the final sample substantially exceeded this.

## Results

### Descriptive statistics

Descriptive statistics for all Items are provided in Table 1 and for all MEDI-Q indices and validation measures in Table 2. The MEDI-Q Total Score ranged from 0 to 125, with a mean of 17.87 (*SD* = 13.13), somewhat higher than values reported in the Italian (*M* = 14.94, *SD* = 12.73 [21]) and English validation studies (*M* = 12.43, *SD* = 11.27 [22]), with effect sizes in the small-to-medium range (*d* = 0.23 and 0.45, respectively). Mean scores were considerably higher than those observed in the Indonesian validation (*M* = 8.79, *SD* = 9.11 [23]; *d* = 0.82). The mean MS was 8.33 (*SD* = 4.34), MSD was 2.00 (*SD* = 0.70), and MESI was 0.54 (*SD* = 0.29). For 24 participants (2.3%), MS equalled zero, rendering MSD and MESI undefined; these cases were treated as missing for all analyses involving these indices. Applying the proposed cut-off of ≥ 20 [21], 377 participants (36.4%) exceeded the threshold. This figure should not be interpreted as a population-level prevalence estimate, as the cut-off has not been empirically validated against clinical diagnosis in the present or prior samples, and the self-selected community sample may over-represent individuals with pronounced menstrual symptoms.

**Table 1:** Item Statistics and Corrected Item-Total Correlations for the German MEDI-Q (*N* = 1,035)

Item	Item content	M	SD	*r*_it_
1	Lower abdominal pain	1.99	1.52	0.467
2	Urinary pain	0.07	0.37	0.199
3	Pain at defecation	0.29	0.77	0.293
4	Muscle or osteoarticular pain	0.75	1.21	0.416
5	Breast tenderness or widespread swelling sensation	0.73	1.14	0.346
6	Nausea	0.40	0.95	0.348
7	Headache	0.62	1.12	0.295
8	Digestive problems	0.76	1.26	0.456
9	Diarrhoea	0.92	1.26	0.350
10	Constipation	0.17	0.62	0.238
11	Discomfort due to vaginal bleeding	1.61	1.70	0.362
12	Feeling of being impure	0.83	1.38	0.348
13	Sadness	1.27	1.44	0.565
14	Emotional lability	1.30	1.44	0.559
15	Irritability or anger	1.09	1.33	0.539
16	Impulsiveness	0.16	0.62	0.288
17	Anxiety	0.41	0.92	0.422
18	Increased appetite	0.75	1.18	0.297
19	Decreased appetite	0.23	0.79	0.253
20	Insomnia	0.28	0.82	0.352
21	Hypersomnia	0.67	1.24	0.519
22	Fatigue	1.26	1.51	0.590
23	Decreased sexual drive	0.62	1.29	0.216
24	Concentration impairment	0.51	1.12	0.470
25	Pain during sexual intercourse	0.20	0.72	0.239
	Total Scale	17.87	13.13	

*M*  mean item score (range 0–5), *SD*  standard deviation, *r*_it_ corrected item-total correlation. Cronbach’s α = 0.839, McDonald’s ω = 0.844. The complete German version of the MEDI-Q is provided in Supplementary Material 1, Appendix A

**Table 2:** Means, Standard Deviations, Internal Consistency (diagonal), and Intercorrelations (off-diagonal) Among All Study Variables

Variable	M	SD	1	2	3	4	5	6	7	8	9	10	11	12	13
1. MEDI-Q Total Score	17.87	13.13	*0.839/0.844*												
2. MEDI-Q MS	8.33	4.34	0.884**	*0.789/0.790*											
3. MEDI-Q MSD	2.00	0.70	0.729**	0.412**	*—*										
4. MEDI-Q MESI	0.54	0.29	0.000	−0.072*	0.110**	*0.721/0.717*									
5. Age	31.63	7.07	−0.138**	−0.159**	−0.070*	−0.110**	*—*								
6. DASS-21 Total Score	13.07	10.37	0.361**	0.370**	0.224**	−0.159**	−0.083*	*0.913/0.916*							
7. DASS Depression	4.18	4.14	0.269**	0.275**	0.163**	−0.137**	−0.077*	0.882**	*0.871/0.874*						
8. DASS Anxiety	2.79	3.15	0.322**	0.328**	0.200**	−0.085*	−0.130**	0.787**	0.565**	*0.711/0.720*					
9. DASS Stress	6.10	4.71	0.343**	0.350**	0.214**	−0.172**	−0.031	0.900**	0.685**	0.568**	*0.863/0.865*				
10. PAF10 Total Score	27.77	10.05	0.521**	0.505**	0.392**	−0.304**	0.037	0.534**	0.437**	0.432**	0.505**	*0.862/0.857*			
11. PAF10 Physiological	16.11	6.38	0.476**	0.456**	0.367**	−0.240**	0.055	0.390**	0.303**	0.349**	0.360**	0.910**	*0.780/0.780*		
12. PAF10 Psychological	11.66	5.00	0.440**	0.432**	0.317**	−0.304**	0.004	0.576**	0.492**	0.422**	0.555**	0.848**	0.553**	*0.883/0.882*	
13. Global Rating	5.50	2.37	0.555**	0.502**	0.490**	0.085*	−0.040	0.278**	0.226**	0.262**	0.240**	0.439**	0.432**	0.331**	*—*

*M*  mean, *SD*  standard deviation, *MS*  Menstrual Symptoms, *MSD*  Menstrual Symptom Distress, *MESI*  Menstrual Specificity Index, *DASS*  Depression Anxiety Stress Scales-21, *PAF10*  Premenstrual Assessment Form. For MEDI-Q Total Score, MS, and Age: *N* = 1,035. For MEDI-Q MSD and MESI: *n* = 1,011. For DASS-21 Total and DASS subscales: *n* = 936. For PAF10 variables: *n* = 891. For Global Rating: *n* = 880. Diagonal entries report internal consistency estimates (Cronbach’s α / McDonald’s ω) for multi-item scales; a dash (—) indicates inapplicability. No α is reported for MEDI-Q MSD, as it is a derived index (Total Score / MS) rather than a summated scale; α is likewise not applicable for Age and Global Rating, as both are single-item measures. Off-diagonal entries are Pearson correlation coefficients and are not directly comparable to the diagonal reliability coefficients

* *p* < .05. ** *p* < .01

### Reliability

Cronbach’s α for the MEDI-Q Total Score was 0.839, and McDonald’s ω was 0.844, confirming H1a. Corrected item-total correlations ranged from 0.199 (Item 2: urinary pain) to 0.590 (Item 22: fatigue). Item-level statistics are reported in Table 1. A subsample of *n* = 20 participants completed the MEDI-Q a second time (interval: *M* = 180.50 days, *SD* = 36.87, range 144–280 days), yielding a test-retest reliability coefficient (ICC) for the MEDI-Q Total Score of 0.823 (95% CI [0.61, 0.93]), supporting H1b. Given the small subsample size (*n* = 20) and the naturalistic six-month retest interval, this estimate is underpowered and should be considered exploratory rather than a precise measure of stability.

### Convergent validity

All convergent validity hypotheses were supported (see Table 2). As expected by construction, the MEDI-Q Total Score and MESI were uncorrelated (*r* = .000). This zero correlation is not an empirical finding but a mathematical necessity: the MESI is calculated as the ratio of menstrual-phase distress to total cross-phase distress, and this normalisation procedure removes variance attributable to overall symptom burden by construction. The absence of correlation therefore reflects the scoring design rather than empirical independence between overall severity and phase-specificity. All four MEDI-Q indices correlated negatively with age (Total Score: *r* = − .138; MS: *r* = − .159; MSD: *r* = − .070; MESI: *r* = − .110; all *p*s ≤ 0.026), confirming H2.

Total Score, MS, and MSD were significantly and positively associated with all three DASS-21 subscales (Depression, Anxiety, Stress; *r*s = 0.163–0.350, all *p*s < 0.001), supporting H3. Total Score (*r* = .521), MS (*r* = .505), and MSD (*r* = .392) were all positively associated with PAF10 scores (all *p*s < 0.001), supporting H4. MESI showed a significant negative correlation with PAF10 (*r* = − .304, *p* < .001), supporting H5.

Total Score (*r* = .555), MS (*r* = .502), and MSD (*r* = .490) were substantially associated with the global symptom rating (all *p*s < 0.001), supporting H6a–c. The MESI–global rating association was markedly weaker (*r* = .085, *p* = .013), and Steiger’s Z-tests confirmed it was significantly smaller than those for Total Score (*Z* = 11.35), MS (*Z* = 9.41), and MSD (*Z* = 10.02; all *p*s < 0.001), supporting H6d.

### Structural validity

Three competing CFA models were estimated using maximum likelihood: a 1-factor model, a 2-factor model, and a 5-factor model based on the theoretical domain structure of Vannuccini et al. [21]. Fit indices are presented in Table 3, standardised factor loadings in Table 4.

**Table 3:** Confirmatory Factor Analysis: Model Fit Indices for Three Competing Factor Models of the German MEDI-Q

Model	χ²	df	χ²/df	CFI	TLI	RMSEA [90% CI]	AIC
1-Factor	1609.00	275	5.85	0.725	0.700	0.068 [0.065, 0.072]	1709.00
2-Factor	1327.91	274	4.85	0.783	0.762	0.061 [0.058, 0.064]	1429.91
5-Factor	1130.28	265	4.27	0.822	0.798	0.056 [0.053, 0.060]	1250.28

*CFI*  comparative fit index, *TLI*  Tucker-Lewis index, *RMSEA*  root mean square error of approximation, *AIC*  Akaike information criterion. Acceptable fit: CFI/TLI ≥ 0.90, RMSEA ≤ 0.08 [45]. Chi-square difference tests indicated significantly better fit for the 2-factor over the 1-factor model (Δχ²(1) = 281.08, *p* < .001) and for the 5-factor over the 2-factor model (Δχ²(9) = 197.64, *p* < .001). All models were estimated using maximum likelihood. *N* = 1,035

**Table 4:** Standardized Factor Loadings for Three Competing CFA Models of the German MEDI-Q

Item	1-Factor	2-Factor		5-Factor				
	F1	Physiological	Psychological	Pain	Gastro-intestinal Symptoms	Discomfort	Psychological/ Cognitive	Other Physiological Changes
1. Lower abdominal pain	0.483	0.528		0.562				
2. Urinary pain	0.208	0.228		0.250				
3. Pain at defecation	0.305	0.332		0.378				
4. Muscle/ osteoarticular pain	0.456	0.469		0.499				
5. Breast tenderness/ widespread swelling sensation	0.375	0.371				0.351		
6. Nausea	0.353	0.395			0.471			
7. Headache	0.315	0.331		0.338				
8. Digestive problems	0.479	0.498			0.588			
9. Diarrhoea	0.364	0.410			0.502			
10. Constipation	0.254	0.247			0.264			
11. Discomfort due to vaginal bleeding	0.362	0.380				0.603		
12. Feeling of being impure	0.352	0.354				0.601		
13. Sadness	0.645		0.717				0.719	
14. Emotional lability	0.645		0.768				0.768	
15. Irritability/ anger	0.625		0.725				0.725	
16. Impulsiveness	0.348		0.386				0.385	
17. Anxiety	0.480		0.509				0.508	
18. Increased appetite	0.331	0.315						0.310
19. Decreased appetite	0.266	0.279						0.245
20. Insomnia	0.383	0.393						0.399
21. Hypersomnia	0.572	0.603						0.685
22. Fatigue	0.649	0.679						0.768
23. Decreased sexual drive	0.210	0.232						0.212
24. Concentration impairment	0.519		0.438				0.436	
25. Pain during sexual intercourse	0.230	0.264		0.278				

Only loadings on the assigned factor are shown for each model. All loadings significant at *p* < .001

None of the models achieved acceptable CFI or TLI (≥ 0.90). The 5-factor model showed the best overall fit (CFI = 0.822, TLI = 0.798, RMSEA = 0.056, AIC = 1250.28) and was significantly superior to both the 2-factor and 1-factor models. Factor loadings were strongest for mood-related items (sadness, emotional lability, irritability; λ = 0.625–0.768) and weakest for low-frequency somatic items such as urinary pain (Item 2) and pain during intercourse (Item 25; λ = 0.208–0.278). In the 5-factor model, high interfactor correlations, particularly between Pain and Gastrointestinal factors (*r* = .900), indicated substantial overlap between theoretically proposed domains.

## Discussion

The present study examined the psychometric properties of a German-language version of the MEDI-Q in a large community sample of German-speaking women. The findings provide broad support for the reliability and convergent validity of the German MEDI-Q. Structural validity, however, could not be established: no tested factor model achieved acceptable fit, and subscale-level interpretation is not empirically warranted at this stage.

Internal consistency of the German MEDI-Q Total Score was satisfactory (α = 0.839), closely replicating values from the Italian and English validations (α = 0.85 and 0.84, respectively [21, 22]), and confirming H1a. The recently published Indonesian validation demonstrated markedly higher internal consistency (α = 0.972 [23]), though this likely reflects its small hospital-based convenience sample (*N* = 53) rather than superior instrument performance. This cross-linguistic replicability suggests that the 25 items assess a coherent underlying construct of menstrual distress across cultural contexts. Corrected item-total correlations were satisfactory for most items; weaker discrimination was observed for low-frequency somatic symptoms such as urinary pain (Item 2) and pain during intercourse (Item 25), consistent with findings from the English validation [22] and attributable to restricted variance in rarely endorsed items.

Test-retest reliability (ICC = 0.823) was below the values of 0.95 reported in prior studies, which employed retest intervals of 7–14 days, but is interpreted as a conservative lower bound given the naturalistic six-month interval of the present study. Genuine intraindividual changes in menstrual symptom burden are plausible over such a period, making direct comparison inherently asymmetric. Taken together, the reliability findings are consistent with the conceptualisation of menstrual distress as a relatively stable characteristic, though this interpretation requires replication with adequately powered test-retest designs.

The present study provides the first CFA examination of the MEDI-Q across all four language versions. Neither prior validation included confirmatory factor analyses. The present findings should therefore be interpreted as an initial, hypothesis-generating contribution rather than a definitive evaluation against an established benchmark. Among the three competing models, the theoretically proposed 5-factor structure demonstrated the best relative fit and was significantly superior to both the 2-factor and 1-factor alternatives. However, as none of the models reached conventional fit thresholds, these comparisons do not constitute evidence of structural validity. No empirically supported subscale structure can be established on the basis of the present data, and the five-domain organisation proposed by the instrument developers remains theoretically motivated but structurally unconfirmed. That none of the models achieved acceptable fit by conventional CFI/TLI criteria (≥ 0.90) is not unexpected given the instrument’s characteristics: the MEDI-Q covers a deliberately heterogeneous symptom spectrum, and high interfactor correlations, particularly between the Pain and Gastrointestinal factors, reflect genuine symptom co-occurrence rather than a measurement artefact. The ordinal item distribution and restricted variance in low-frequency items (e.g., urinary pain, pain during intercourse) likely contributed to suboptimal model fit, as is commonly observed under these conditions regardless of estimation method.

Crucially, suboptimal fit in CFA does not preclude the validity or clinical utility of a total score. The MEDI-Q Total Score demonstrated satisfactory internal consistency, adequate convergent validity, and meaningful associations with external criteria across all four validation studies. These findings support its use as a global index of menstrual distress burden. However, the present results caution against treating the five subscales as psychometrically independent constructs in the absence of further structural validation. Future studies should explore alternative structural specifications; a bifactor model attempted in the present data proved non-convergent, likely due to limited indicator counts on some group factors, highlighting that such approaches may require a revised or expanded item pool. Exploratory structural equation modelling represents a further promising avenue for future investigation. From a clinical standpoint, the MEDI-Q Total Score shows promise as an assessment tool in gynaecological outpatient settings, where brief, standardised measurement of menstrual distress could inform clinical decision-making. The cut-off of ≥ 20 was empirically derived and clinically validated in the Italian original (AUC = 0.90; Vannuccini et al., 2021), providing a theoretically grounded starting point. However, its applicability to German-speaking populations requires independent validation against clinically confirmed diagnoses before sensitivity and specificity can be established, and it should therefore be treated as a provisional research benchmark rather than a validated screening threshold. Empirical validation in clinically defined samples, including individuals with endometriosis, primary dysmenorrhoea, or premenstrual dysphoric disorder, remains an important next step.

All convergent validity hypotheses were supported. In line with H2, all four MEDI-Q indices correlated negatively with age, replicating the Italian validation [21] and consistent with the broader literature on hormonal changes across the reproductive lifespan [32, 33]. The negative association of MESI with age is a novel finding, suggesting that menstrual phase-specificity of symptom exacerbation also decreases with age, possibly reflecting a blurring of cycle-phase distinctions approaching the perimenopause transition.

Consistent with H3, Total Score, MS, and MSD were positively associated with all three DASS-21 subscales (small-to-medium effects), replicating prior MEDI-Q validations [21, 22] and consistent with the broader literature [12–15, 34]. The use of the DASS-21 rather than the BSI employed in prior studies extends the nomological network of the MEDI-Q to an additional validated measure of psychological distress.

The large associations between Total Score, MS, and MSD and the global symptom rating (H6a–c) confirm convergent validity against a face-valid criterion. The markedly weaker association for the MESI (H6d) supports its interpretive distinctiveness: the MESI captures phase-specificity rather than overall burden, a distinction with direct implications for clinical application of the instrument.

### Strengths, limitations, and future directions

The present study has several notable strengths. The sample substantially exceeds those of all prior validation studies and affords high statistical power. Coverage of Germany, Austria, and Switzerland supports applicability across the major German-speaking countries. Wide ranges in menarcheal age, cycle length, and duration indicate broad menstrual variability, and the rigorous translation procedure ensures linguistic and conceptual equivalence. The inclusion of a global symptom severity rating extends the nomological validation beyond measures used in prior studies.

Several limitations should be acknowledged. The online convenience and snowball sampling approach may have introduced self-selection bias, with individuals with more salient menstrual complaints potentially over-represented, inflating mean scores relative to the population. Relatedly, the low rate of hormonal contraceptive use (12.2%) is notably below the estimated prevalence of approximately 38% among German women of reproductive age [48], suggesting the sample over-represents naturally cycling women. Sensitivity analyses excluding hormonal contraceptive users (*n* = 909) yielded substantively identical results: internal consistency was α = 0.842, ω = 0.846, the proportion exceeding the proposed cut-off was 36.5% (vs. 35.7% in contraceptive users; χ²(1) = 0.031, *p* = .860), and the pattern of convergent validity correlations was replicated in direction and magnitude. Independent-samples t-tests indicated no significant differences between groups on MEDI-Q Total Score (*p* = .116), MS (*p* = .060), or MSD (*p* = .995). A small significant difference emerged for MESI (*p* = .039, *d* = 0.20), with contraceptive users showing slightly higher phase-specificity scores, possibly reflecting altered cycle phase perception under hormonal contraception. These findings suggest that inclusion of hormonal contraceptive users did not materially affect the primary results, though replication in more balanced samples remains warranted. The cross-sectional design precludes causal inference. The test-retest subsample was small (*n* = 20) and the naturalistic six-month retest interval substantially exceeds prior validation studies (7–14 days), limiting the precision of the ICC estimate. The exclusion of women with diagnosed uterine pathology or psychiatric disorders limits generalisability to clinical populations, in which the MEDI-Q may ultimately prove most useful. This exclusion may furthermore have restricted the variance of DASS-21 scores in the present sample, potentially attenuating the observed associations between MEDI-Q indices and psychological distress; the true convergent validity with psychological distress measures may therefore be somewhat underestimated. Empirically validating the proposed cut-off score of 20 in samples with endometriosis, dysmenorrhoea, or premenstrual dysphoric disorder, and investigating measurement invariance across age groups and contraceptive use status, represent important next steps.

## Conclusions

The German MEDI-Q demonstrates satisfactory internal consistency, acceptable test-retest reliability over a naturalistic long-term interval, and adequate convergent validity, replicating the psychometric profile of the Italian, English, and Indonesian versions. It represents a promising instrument for the standardised assessment of menstrual distress in German-speaking research and clinical contexts. No tested factor model achieved acceptable fit, and the MEDI-Q should currently be interpreted as a total score instrument; subscale-level interpretation is not empirically supported pending further structural investigation. With this German validation, the MEDI-Q is now available in four languages, enabling cross-cultural comparison of menstrual distress prevalence, symptom burden, and treatment outcomes.

## Supplementary Information

Supplementary Material 1.

## Data Availability

The datasets used and/or analysed during the current study are available from the corresponding author on reasonable request.

## Abbreviations

AIC: Akaike Information Criterion
AMOS: Analysis of Moment Structures
BSI: Brief Symptom Inventory
BZgA: Bundeszentrale für gesundheitliche Aufklärung
CFA: Confirmatory Factor Analysis
CFI: Comparative Fit Index
CI: Confidence Interval
DASS-21: Depression Anxiety Stress Scales–21
EFA: Exploratory Factor Analysis
ICC: Intraclass Correlation Coefficient
ITC: International Test Commission
MDQ: Menstrual Distress Questionnaire [16]
MEDI-Q: Menstrual Distress Questionnaire
MESI: Menstrual Specificity Index
MS: Menstrual Symptoms
MSD: Menstrual Symptom Distress
PAF10: Premenstrual Assessment Form–10
RMSEA: Root Mean Square Error of Approximation
SD: Standard Deviation
SPSS: Statistical Package for the Social Sciences
TLI: Tucker–Lewis Index
