# Supplementary material for: The Menstrual Distress Questionnaire (MEDI-Q): reliability and validity of the German version
Source: BMC Womens Health. 2026 Jul 14;26:357. doi: 10.1186/s12905-026-04608-7 (PMC13366723; doi:10.1186/s12905-026-04608-7)
Supplement: Supplementary file 1 — Supplementary Material 1. [file 12905_2026_4608_MOESM1_ESM.pdf]

Menstrual Distress Questionnaire (MEDI-Q), deutsche Version

Instruktion - Bitte lesen Sie die Liste der angegebenen Symptome sorgfältig durch. Bitte beantworten Sie Frage A für jedes Symptom, das Sie während Ihrer Menstruationszyklen in den letzten 12 Monaten verspürt haben. Wenn Sie ein bestimmtes Symptom nicht erlebt haben, antworten Sie bitte mit "Nein" und fahren Sie mit dem nächsten Symptom auf der Liste fort. Wenn Sie jedoch ein bestimmtes Symptom während Ihrer Menstruationszyklen verspürt haben, beantworten Sie bitte auch die Fragen B, C und D zu den Auswirkungen dieses Symptoms auf Ihr Funktionieren und Ihre Lebensqualität.

**A. Hatten Sie im vergangenen Jahr an den Tagen Ihrer Menstruation …**				**Wenn Sie dieses Symptom hatten, in welchem Ausmaß hat es Ihre Lebensqualität, Ihre Freizeit- oder Arbeitsaktivitäten oder Ihre sozialen Beziehungen beeinträchtigt …**													
				**B. … an den Tagen der Menstruation?**				**C. … in der prämenstruellen Phase (in den 7 Tagen vor Beginn der Menstruation)?**					**D. … an anderen Tagen (außerhalb der Menstruations-/Prämenstruationsphase)?**				
	Ja, mehr als die Hälfte der Zeit, in der ich meine Periode hatte	Ja, weniger als die Hälfte der Zeit, in der ich meine Periode hatte	Nein *(zum nächsten Punkt gehen)*	gar nicht	wenig	ziemlich	sehr stark	gar nicht	wenig	ziemlich	sehr stark	Ich hatte dieses Symptom noch nie in der prämenstruellen Phase	gar nicht	wenig	ziemlich	sehr stark	Ich hatte dieses Symptom noch nie außerhalb der Menstruations-/ Prämenstruations-phase
1. … Unterleibsschmerzen?	☐	☐	☐	☐	☐	☐	☐	☐	☐	☐	☐	☐	☐	☐	☐	☐	☐
2. … Schmerzen beim Wasserlassen?	☐	☐	☐	☐	☐	☐	☐	☐	☐	☐	☐	☐	☐	☐	☐	☐	☐
3. … Schmerzen beim Stuhlgang?	☐	☐	☐	☐	☐	☐	☐	☐	☐	☐	☐	☐	☐	☐	☐	☐	☐
4. … Muskel-, Knochen-, Gelenkschmerzen?	☐	☐	☐	☐	☐	☐	☐	☐	☐	☐	☐	☐	☐	☐	☐	☐	☐
5. … ein Spannungsgefühl in den Brüsten oder eine diffuse Schwellung?	☐	☐	☐	☐	☐	☐	☐	☐	☐	☐	☐	☐	☐	☐	☐	☐	☐
6. … Übelkeit?	☐	☐	☐	☐	☐	☐	☐	☐	☐	☐	☐	☐	☐	☐	☐	☐	☐
7. … Kopfschmerzen?	☐	☐	☐	☐	☐	☐	☐	☐	☐	☐	☐	☐	☐	☐	☐	☐	☐
8. … Verdauungsbeschwerden (lange Verdauung, Sodbrennen, störendes Völlegefühl nach den Mahlzeiten …)?	☐	☐	☐	☐	☐	☐	☐	☐	☐	☐	☐	☐	☐	☐	☐	☐	☐
9. … Durchfall?	☐	☐	☐	☐	☐	☐	☐	☐	☐	☐	☐	☐	☐	☐	☐	☐	☐
10. … Verstopfung?	☐	☐	☐	☐	☐	☐	☐	☐	☐	☐	☐	☐	☐	☐	☐	☐	☐
11. … Unwohlsein oder Beschwerden aufgrund der vaginalen Blutungen (Angst vor Flecken/ Geruch, Unbehagen durch Binden/Tampons, Schwierigkeiten oder Verlegenheit beim Geschlechtsverkehr …)?	☐	☐	☐	☐	☐	☐	☐	☐	☐	☐	☐	☐	☐	☐	☐	☐	☐
12. … sich unrein gefühlt?	☐	☐	☐	☐	☐	☐	☐	☐	☐	☐	☐	☐	☐	☐	☐	☐	☐
13. … das Gefühl, übermäßig traurig zu sein (leichtes Anfangen zu weinen, geringer Antrieb Dinge zu tun, Interessensverlust an üblichen Aktivitäten …)?	☐	☐	☐	☐	☐	☐	☐	☐	☐	☐	☐	☐	☐	☐	☐	☐	☐

**A. Hatten Sie im vergangenen Jahr an den Tagen Ihrer Menstruation …**				**Wenn Sie dieses Symptom hatten, in welchem Ausmaß hat es Ihre Lebensqualität, Ihre Freizeit- oder Arbeitsaktivitäten oder Ihre sozialen Beziehungen beeinträchtigt …**													
				**B. … an den Tagen der Menstruation?**				**C. … in der prämenstruellen Phase (in den 7 Tagen vor Beginn der Menstruation)?**					**D. … an den anderen Tagen (außerhalb der Menstruations-/Prämenstruationsphase)?**				
	Ja, mehr als die Hälfte der Zeit, in der ich meine Periode hatte	Ja, weniger als die Hälfte der Zeit, in der ich meine Periode hatte	Nein *(zum nächsten Punkt gehen)*	gar nicht	wenig	ziemlich	sehr stark	gar nicht	wenig	ziemlich	sehr stark	Ich hatte dieses Symptom noch nie in der prämenstruellen Phase	gar nicht	wenig	ziemlich	sehr stark	Ich hatte dieses Symptom noch nie außerhalb der Menstruations-/ Prämenstruations-phase
14. … sich emotional labil gefühlt (Stimmungsschwankungen als Reaktion auf kleinste Auslöser …)?	☐	☐	☐	☐	☐	☐	☐	☐	☐	☐	☐	☐	☐	☐	☐	☐	☐
15. … sich reizbar oder irritierbar gefühlt (nervös sein, unerwartete Ereignisse, Menschen oder Situationen nicht ertragen können, sich leicht ärgern …)?	☐	☐	☐	☐	☐	☐	☐	☐	☐	☐	☐	☐	☐	☐	☐	☐	☐
16. … sich impulsiv gefühlt (Drang zu Handeln ohne nachzudenken oder zu planen …)?	☐	☐	☐	☐	☐	☐	☐	☐	☐	☐	☐	☐	☐	☐	☐	☐	☐
17. … sich ängstlich gefühlt (aufgeregt, angespannt, übermäßig unsicher oder unentschlossen; Angst, dass jeden Moment etwas Schlimmes passieren könnte …)?	☐	☐	☐	☐	☐	☐	☐	☐	☐	☐	☐	☐	☐	☐	☐	☐	☐
18. … zu viel Appetit (übermäßiges Verlangen zu essen, Verlust der Kontrolle über das Essen …)?	☐	☐	☐	☐	☐	☐	☐	☐	☐	☐	☐	☐	☐	☐	☐	☐	☐
19. … geringen Appetit?	☐	☐	☐	☐	☐	☐	☐	☐	☐	☐	☐	☐	☐	☐	☐	☐	☐
20. … Schlaflosigkeit?	☐	☐	☐	☐	☐	☐	☐	☐	☐	☐	☐	☐	☐	☐	☐	☐	☐
21. … übermäßige Schläfrigkeit (Schlafen am Tag, morgens nicht aufstehen können …)?	☐	☐	☐	☐	☐	☐	☐	☐	☐	☐	☐	☐	☐	☐	☐	☐	☐
22. … übermäßige Müdigkeit (schlapp, wenig Energie …)?	☐	☐	☐	☐	☐	☐	☐	☐	☐	☐	☐	☐	☐	☐	☐	☐	☐
23. … geringes sexuelles Verlangen (vermindertes Verlangen nach Geschlechtsverkehr, geringe sexuelle Fantasien …)?	☐	☐	☐	☐	☐	☐	☐	☐	☐	☐	☐	☐	☐	☐	☐	☐	☐
24. … Konzentrationsschwierigkeiten?	☐	☐	☐	☐	☐	☐	☐	☐	☐	☐	☐	☐	☐	☐	☐	☐	☐

25. Hatten Sie im vergangenen Jahr Geschlechtsverkehr mit vaginaler Penetration?	☐ Ja		☐ Nein *(Fragebogen beenden) (0)*		
25A. Hatten Sie im vergangenen Jahr an Tagen Ihrer Menstruation, jemals Schmerzen beim Geschlechtsverkehr mit vaginaler Penetration?	☐ Ja, mehr als die Hälfte der Zeit, in der ich meine Periode hatte, hatte ich Schmerzen bei der vaginalen Penetration. (2)<br>☐ Ja, weniger als die Hälfte der Zeit, in der ich meine Periode hatte, hatte ich Schmerzen bei der vaginalen Penetration (1)<br>☐ Nein, ich hatte nie Schmerzen bei der vaginalen Penetration. *(Fragebogen beenden) (0)*<br>☐ Ich hatte nie Geschlechtsverkehr an Tagen der Periode, weil ich dann zu starke Schmerzen hatte. (2)<br>☐ Ich hatte aus anderen Gründen als Schmerzen, noch nie Geschlechtsverkehr mit vaginaler Penetration an Tagen der Periode. *(Fragebogen beenden) (0)*				
25B. Wie sehr hat der Schmerz bei der vaginalen Penetration (oder das Vermeiden von vaginaler Penetration) **an den Tagen Ihrer Menstruation**, Ihre Lebensqualität, Ihre Freizeit-, Arbeitsaktivitäten oder sozialen Kontakte beeinträchtigt?	☐ gar nicht	☐ wenig	☐ ziemlich	☐ sehr stark	
25C. Wie sehr hat der Schmerz bei der vaginalen Penetration (oder das Vermeiden von vaginaler Penetration) **in der prämenstruellen Phase** (in den 7 Tagen vor Beginn der Menstruation), Ihre Lebensqualität, Ihre Freizeit-, Arbeitsaktivitäten oder sozialen Kontakte beeinträchtigt?	☐ gar nicht	☐ wenig	☐ ziemlich	☐ sehr stark	☐ Ich hatte dieses Symptom noch nie in der prämenstruellen Phase.
25D. Wie sehr hat der Schmerz bei der vaginalen Penetration (oder das Vermeiden von vaginaler Penetration) **an den anderen Tagen** (außerhalb der menstruellen/ prämenstruellen Phase), Ihre Lebensqualität, Ihre Freizeit-, Arbeitsaktivitäten oder sozialen Kontakte beeinträchtigt?	☐ gar nicht	☐ wenig	☐ ziemlich	☐ sehr stark	☐ Ich hatte dieses Symptom noch nie außerhalb der Menstruations-/ Prämenstruationsphase.

Citation

Görlich, Y & Richter, E. (2026). The Menstrual Distress Questionnaire (MEDI-Q): Reliability and Validity of the German Version. *BMC Women's Health.* https://doi.org/10.1186/s12905-026-04608-7

Original Italian version:

Vannuccini, S., Rossi, E., Cassioli, E., Cirone, D., Castellini, G., Ricca, V., & Petraglia, F. (2021). Menstrual Distress Questionnaire (MEDI-Q): a new tool to assess menstruation-related distress. *Reproductive Biomedicine Online, 43*(6), 1107–1116. https://doi.org/10.1016/j.rbmo.2021.08.029

English version:

Cassioli, E., Rossi, E., Melani, G., Faldi, M., Rellini, A. H., Wyatt, R. B., Oester, C., Vannuccini, S., Petraglia, F., Ricca, V., & Castellini, G. (2023). The Menstrual Distress Questionnaire (MEDI-Q): Reliability and validity of the English version. *Gynecological Endocrinology, 39*(1). https://doi.org/10.1080/09513590.2023.2227275

MEDI-Q - Auswertungsanleitung

SCHRITT 1 - Berechnung der Unterpositionen

- Für jede Position (1-25):

1. Vergeben Sie eine Häufigkeitszahl (A) auf der Grundlage der Antwort auf Frage A, wie folgt:

**2** Ja, mehr als die Hälfte der Zeit, in der ich meine Periode hatte	**1** Ja, weniger als die Hälfte der Zeit, in der ich meine Periode hatte	**0** Nein

Für Item 25 sind die in Klammern angegebenen Häufigkeitsangaben zu verwenden.
Bei Items mit einem Häufigkeitswert von 0 ist es möglich, direkt einen Endwert von 0 zu vergeben.

2. Vergeben Sie eine Punktzahl für Menstruationsbeschwerden (B) auf der Grundlage der Antwort auf Frage B wie folgt:

**0** gar nicht	**1** wenig	**2** ziemlich	**3** sehr stark

3. Vergeben Sie eine Punktzahl für prämenstruelles Leiden (C) auf der Grundlage der Antwort auf Frage C wie folgt:

**0** gar nicht *oder* Ich hatte dieses Symptom nie in der prämenstruellen Phase	**1** wenig	**2** ziemlich	**3** sehr stark

4. Vergeben Sie eine Punktzahl für intermenstruelles Leiden (D) auf der Grundlage der Antwort auf Frage D wie folgt:

**0** gar nicht *oder* Ich hatte dieses Symptom nie außerhalb der Menstruations-/Prämenstruationsphase	**1** wenig	**2** ziemlich	**3** sehr stark

5. Berechnen Sie den Δ-Belastungswert, indem Sie D von B abziehen: Δ-Belastungswert = B - D
   Wenn das Ergebnis negativ ist, verwenden Sie stattdessen den Wert 0.

6. Berechnen Sie die endgültige Gesamtpunktzahl auf der Grundlage der Häufigkeitszahl (A) und des Δ-Belastungswertes wie folgt:

		Häufigkeitswert (Unterpunkt A)		
		**0**	**1**	**2**
Δ Belastungswert (B - D)	**0**	0	0	0
	**1**	0	1	2
	**2**	0	2	4
	**3**	0	3	5

Eine einfache Möglichkeit, dies zu erreichen, besteht darin, die Häufigkeitszahl mit dem Δ-Belastungswert zu multiplizieren, und wenn das Ergebnis der Multiplikation gleich 6 ist, stattdessen den Wert 5 zu verwenden.

SCHRITT 2 - Berechnung des Indizes für Menstruationsbeschwerden

1. *MEDI-Q-Gesamtscore*: Addieren Sie alle im vorherigen Schritt berechneten Item-Punkte. Dieser Wert reicht von einem Minimum von 0 bis zu einem Maximum von 125.
2. *MEDI-Q Menstruationssymptome (MS)*: die Gesamtzahl der Items, für die der Endwert größer als Null ist. Sie gibt die Anzahl der Symptome an, die während der Menstruationstage größere Beschwerden verursachen als während der Zeit zwischen den Menstruationen. Dieser Wert reicht von einem Minimum von 0 bis zu einem Maximum von 25.
3. *MEDI-Q Menstruationssymptom-Belastung (MSD)*: wird berechnet, indem der MEDI-Q-Gesamtscore durch MEDI-Q MS geteilt wird. Er gibt den durchschnittlichen Leidensdruck an, der durch Symptome verursacht wird, bei denen der Leidensdruck während der Menstruationstage im Vergleich zur Zeit zwischen den Menstruationen zunimmt. Dieser Wert reicht von einem Minimum von 0 bis zu einem Maximum von 5.
4. *MEDI-Q Menstruationsspezifitätsindex (MESI)*: gibt den Anteil der Symptome an, bei denen der Leidensdruck während der Menstruationstage im Vergleich zur prämenstruellen und intermenstruellen Phase zunimmt. Er wird berechnet, indem die Anzahl der Items gezählt wird, bei denen Punktwert B größer ist als Punktwert C und Punktwert D, und diese Zahl durch MEDI-Q MS geteilt wird. Dieser Wert liegt zwischen 0 und 1, wobei 0 anzeigt, dass alle Menstruationssymptome auch in der prämenstruellen Phase den gleichen Leidensdruck verursachen, während 1 anzeigt, dass alle Symptome in der Menstruationsphase einen größeren Leidensdruck verursachen als während der prämenstruellen Phase.
